# Communications

**Published:** 1875-02

**Authors:** 


*Communications Received since the Publication of the January No.*

Odontolithos, by T. Curtis Smith, M.D., Middleport, Ohio; A Case of Painless Labor, by Swan M. Burnett, M.D., Knoxville, Tenn.; Several Valuable Articles by J. B. Mattison, M.D., Chester, N. J.; Is the White Blood Corpuscle and the Pus Corpuscle Identical? by T. Curtis Smith, M.D., Middleport, Ohio.

Friends will please forward their communications as soon as possible for the April number.

